# Impact of ethnicity and antihyperglycemic medications on dementia incidence in older adults with type 2 diabetes

**DOI:** 10.1530/EC-25-0593

**Published:** 2026-02-20

**Authors:** Elena Chertok Shacham, Sireen Sharif, Muhammad Kadah, Snait Ayalon

**Affiliations:** ^1^ Endocrinology Unit, Haemek Medical Center, Afula, Israel; ^2^ Faculty of Medicine, Technion – Israel Institute of Technology, Haifa, Israel; ^3^ Statistics Department, Haemek Medical Center, Afula, Israel; ^4^ Internal Medicine, Haemek Medical Center, Afula, Israel; ^5^ Research Authority, Emek Medical Center, Afula, Israel

**Keywords:** diabetes, aging

## Abstract

**Background:**

Type 2 diabetes increases the risk of cognitive decline and dementia. Data on ethnic differences in dementia prevalence among patients with diabetes remain limited.

**Methods:**

We conducted a retrospective matched case–control study of Clalit Health Services members aged 60 or older. Matching (1:3) was based on age, sex, language, and socioeconomic status. Ethnicity was determined by spoken language or place of birth. Prescriptions and refills for antidiabetic medications were recorded for the two years preceding the index date. Clinical, anthropometric, and comorbidity data were analyzed.

**Results:**

During follow-up, 39% of participants were diagnosed with dementia over a maximum observation period of 20 years (crude proportion). However, given a median follow-up of 9.6 years, dementia incidence was primarily evaluated using time-specific competing-risk analyses. When accounting for death as a competing event, the 10-year cumulative incidence of dementia was approximately 9–10%, varying across ethnic groups. However, in multivariable competing-risk models adjusting for age, sex, socioeconomic status, and comorbidities, ethnicity was no longer independently associated with dementia incidence. In matched analyses, the use of SGLT-2 inhibitors and DPP-4 inhibitors was associated with a lower probability of dementia. In time-to-event analyses of dementia-related mortality, treatment with SGLT-2 inhibitors, GLP-1 receptor agonists, and DPP-4 inhibitors was associated with significantly reduced mortality risk.

**Conclusion:**

Ethnic differences in dementia incidence were attenuated after adjustment for demographic and clinical factors. SGLT-2 and DPP-4 inhibitors were associated with a lower risk of dementia, whereas GLP-1 receptor agonists, DPP-4 inhibitors, and SGLT-2 inhibitors were associated with reduced dementia-related mortality.

## Introduction

Dementia, including Alzheimer’s disease and related disorders, greatly affects cognitive, functional, occupational, and social aspects of life, ultimately causing a loss of independence (1).

While age remains the strongest risk factor, research increasingly indicates that ethnicity also significantly influences incidence, risk factors, diagnosis, and dementia outcomes (2). Multiple biological, social, and environmental factors contribute to these disparities, including hypertension, diabetes, and stroke, which are more prevalent in certain minority groups and are strongly linked to dementia (3). Obesity, as measured by the BMI index and central obesity, is related to an increase in waist circumference, which is likely related to the endocrine functions of adipose tissue, mediated by adipokines (4), and could play a significant role, independent of other risk factors. Moreover, postmenopausal metabolic changes, including increased central adiposity and insulin resistance, together with longer life expectancy, may contribute to the higher observed burden of dementia among women (1, 5, 6, 7).

Israel has a population of roughly 9.8 million residents, with 73% of the population of Jewish ethnic origin (approximately 7.1 million), including subgroups such as those of Ethiopian and former Soviet Union descent (8). Around 21% of the population is of Arab descent – Muslim, Christian, or Druze (approximately 2 million) – and about 6% (around 534,000) is classified as ‘other’ (9). It has been found that the prevalence of dementia among the Arab population in Israel is relatively high and even exceeds that in societies with similar education and literacy levels (10). The data about the incidence of dementia in the former Soviet Union are scarce. A higher rate of cognitive impairment, including verbal learning, was observed in FSU immigrants (11).

Diabetes mellitus is associated with a nearly twofold risk of dementia development, according to a solid body of evidence (12). Moreover, the younger age of diabetes was found to be significantly correlated with subsequent development of dementia (13). Growing interest has emerged regarding the association between antihyperglycemic medications and the risk of dementia. In the study of Weinstein *et al.* (14), treatment with insulin was associated with an increased risk of dementia and a greater decline in cognitive function, whereas metformin and sulfonylureas did not reveal such an association.

In addition, systematic reviews and meta-analyses (15, 16) evaluated the impact of various antidiabetic agents on dementia outcomes. SGLT-2 inhibitors and GLP-1 receptor agonists consistently demonstrated dementia risk reduction.

### Research aims

- To investigate the association between ethnicity and the incidence of dementia among adults aged 60 years and older with type 2 diabetes mellitus.
- To evaluate the relationship between various classes of antidiabetic medications and dementia-related survival probability, particularly focusing on new agents, such as GLP-1 receptor agonists and SGLT-2 inhibitors, in comparison with insulin and metformin.

## Methods

This retrospective cohort study included members of Clalit Health Services aged 60 years and older who were not diagnosed with dementia as of January 1, 2004. The observation period spanned from 2004 to 2024. Dementia cases were identified based on a specific ICD code recorded in electronic medical charts. The diagnosis of type 2 diabetes mellitus (DM) was documented according to established ICD codes. Patients were classified into three groups based on the duration of diabetes at the time of dementia diagnosis (or the matched index date for controls). To evaluate the association between antidiabetic treatment and dementia risk, exposure to six classes of antihyperglycemic medications – insulin, GLP-1, SGLT-2, DPP-4 inhibitors, sulfonylureas, and metformin – was examined.

### Definitions and rationale for covariate secretion

Covariates were chosen based on proven evidence from earlier studies, including demographic, metabolic, and vascular factors linked to dementia risk in people with type 2 diabetes.

Demographic variables included age, sex, ethnicity, and socioeconomic status (SES). SES was categorized into low (1–3 points), medium (4–7 points), and high (8–10 points) levels based on the Clalit Health Services area-based socioeconomic index, which combines census data on income, education, and employment within each residential area.

- Clinical covariates included diabetes duration, HbA1c, BMI, hypertension, dyslipidemia, cardiovascular disease (CVD), chronic kidney disease (CKD), and congestive heart failure (CHF). CVD was defined as a composite of ischemic heart disease, stroke, and peripheral vascular disease based on relevant ICD-9 codes. CHF was analyzed separately because it represents a distinct clinical entity.
- SES was defined as described above.
- Demographic variables included age, sex, ethnicity, and smoking status.
- Medication exposure was defined as the use of metformin, insulin, SGLT-2 inhibitors, GLP-1 receptor agonists, DPP-4, sulfonylureas, and statins within the two years preceding the index date.

Cardiovascular disease (CVD) was defined as a composite variable including ischemic heart disease (ICD-9-CM 410–414), stroke or cerebrovascular disease (430–438), and peripheral vascular disease (440.2, 443.9).

CHF was analyzed separately and defined using ICD-9-CM 428.x codes, as it represents a distinct clinical entity.

CKD was identified by ICD-9-CM 585.x and 586.

Hypertension was defined by codes 401–405, and dyslipidemia was defined by 272.0–272.4.

All comorbidities were considered present if at least one diagnosis was recorded in inpatient or outpatient data before the index date.

### Cohort selection and matching process

We identified an initial cohort of 319,843 adults aged 60 years with type 2 DM, who were members of Clalit Medical Services. Patients diagnosed with dementia before January 1, 2004, were excluded. We performed 1:3 matching on age, sex, spoken language, and duration of diabetes. Controls had to be alive and actively insured at the index date (defined as the date of dementia diagnosis for cases or the assigned date for controls). We excluded potential controls who had died before this date to ensure comparability and avoid immortal time bias. After matching, the cohort included 175,249 patients. Our final cohort consisted of data from 150,237 patients.

Ethnicity was categorized as follows:

- Ethiopian origin: based on the country of birth.
- Arab ethnicity: based on the primary spoken language.
- Jewish from the former Soviet Union (FSU): based on the place of birth.
- Other Israeli Jewish: based on Hebrew as the primary language.

### Assessment of treatment compliance

In our study, we used pharmacy refill and dispensation data, which are recorded at the time when a patient actually fills the medication at a Clalit Health Services pharmacy. This reduces the risk of misclassification based on unfilled prescriptions. We assessed adherence using prescription refill patterns over two years before the index date. This exposure window enabled characterization of treatment patterns before the onset of clinical dementia, thus minimizing reverse causality. We classified treatment compliance based on the number of prescriptions dispensed during the exposure period. Patients were classified as compliant, partially compliant, or non-compliant depending on the proportion of expected refills completed. Between six and eight fulfillments were categorized as compliant, between four and five fulfillments were categorized as partially compliant, and fewer than four prescriptions during the two years were categorized as non-compliant. Nevertheless, while the use of dispensation data minimizes bias from unfilled prescriptions, we recognize that adherence beyond pharmacy refills cannot be fully verified.

### Inclusion criteria

- Members of Clalit Health Services aged > 60 years at cohort entry.
- Diagnosis of type 2 DM according to ICD codes.
- No prior diagnosis of dementia before 2004.
- Continuous health insurance coverage for at least one year before the index date.
- Minimum follow-up duration of one year.

### Exclusion criteria

- No type 2 DM diagnosis.
- Control patients who died before their assigned index date.
- Ethnicity not identified according to the demographic data.
- Lack of continuous Clalit Health Services insurance for at least one year before the index date.

### Competing-risk analysis and Fine–Gray models

To quantify the association between covariates and dementia while accounting for the competing risk of death, we applied Fine–Gray subdistribution hazard regression models. We reported subdistribution hazard ratios (sHRs) with 95% confidence intervals (CIs). The primary Fine–Gray model included ethnicity, age, sex, socioeconomic status, and major comorbidities (including cardiovascular disease, CKD, and CHF). Due to computational constraints in the full cohort, multivariable Fine–Gray regression was fitted in a randomly selected subsample of 10,000 participants; this subsample was used only for adjusted association estimates, whereas CIF estimates were derived from the entire cohort.

### Ethical considerations

This study was conducted in accordance with the principles of the Declaration of Helsinki and was approved by an institutional ethics committee. The study utilized fully de-identified data, and no direct patient contact was involved. Therefore, the requirement for informed consent was waived. Data confidentiality and patient privacy were strictly maintained throughout all stages of data handling, storage, and analysis.

### Statistics

Descriptive statistics were used to summarize demographic and clinical characteristics. Group comparisons were performed using the chi-square test for categorical variables and *t*-tests or Mann–Whitney U tests for continuous variables, as appropriate. The propensity score was estimated using the nearest neighbor matching method with a regression model, applying a caliper of 0.02 and a matching ratio of 1:3. Conditional logistic regression was applied to matched data to estimate the association (odds ratios, ORs) between antidiabetic medication use and dementia incidence. To evaluate differences in dementia risk across ethnic groups while accounting for the competing risk of death, we constructed cumulative incidence curves based on the Fine–Gray competing-risk model and compared subdistribution hazards using multivariable Fine–Gray regression.

Missing data were assessed for all covariates before analysis. Variables with a high proportion of missing values (>30%) were excluded from multivariable models. All covariates included in the final analyses had ≤14% missingness.

 Primary analyses were conducted using a complete-case approach. Most missingness reflected structural missingness, defined as values that are not applicable by the study design rather than missing due to incomplete data capture. For example, a dementia diagnosis date is inherently absent for individuals who did not develop dementia, and a death date is absent for individuals who were alive at the end of follow-up. These values were therefore not imputed.

To assess robustness, sensitivity analyses using best-case and worst-case assumptions for missing covariate data were performed and yielded results consistent with the primary analyses.

Cox proportional hazards models were used to assess the relationship between medications, ethnicity, and dementia-related mortality (hazard ratios, HRs). The time-to-event was calculated from the index date to the date of death or the end of follow-up.

Statistical analysis was carried out using R software version 4.1.3. Statistical significance was set at *P* ≤ 0.05.

## Results

Our final cohort included 150,237 adults with type 2 diabetes, comprising 99,998 Jewish patients born in Israel (H), 18,137 Arab patients (A), 29,894 patients born in the former Soviet Union (R), and 2,208 Ethiopian-born patients (E). Patients were followed up from 2004 to 2024 with a median follow-up time of 9.6 years (IQR: 4.8–14.8 years). Overall, 39% of patients developed dementia during the observation period.

### Univariable and multivariable competing-risk analyses

**Figure 1:**
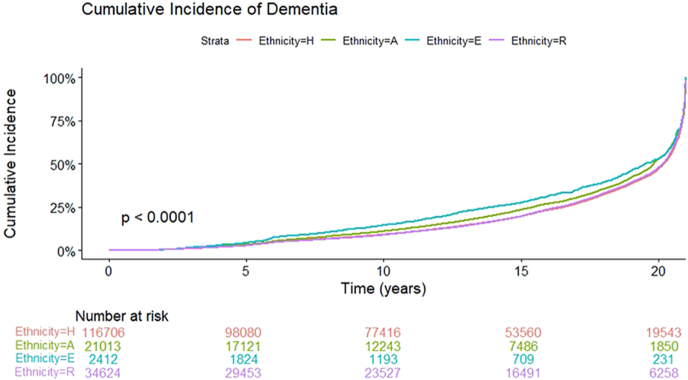
Cumulative incidence of dementia by ethnic group accounting for the competing risk of death.

To estimate dementia incidence while accounting for the competing risk of death, we applied Fine–Gray competing-risk models. The 10-year cumulative incidence of dementia was 13.8% among Ethiopian-born patients (E), 10.5% among Arab patients (A), 8.8% among Israeli-born Jewish patients (H), and 8.6% among former Soviet Union-born patients (R) (Fig. 1).

In univariable Fine–Gray competing-risk models accounting for death as a competing event, dementia incidence differed significantly across ethnic groups, consistent with the cumulative incidence function analyses. Compared with Israeli-born Jewish patients, Ethiopian and Arab patients showed higher unadjusted subdistribution hazards of dementia, whereas former Soviet Union-born patients showed similar or slightly lower hazards.

In contrast, in multivariable Fine–Gray regression adjusting for age, sex, socioeconomic status, cardiovascular disease, CKD, and CHF, ethnicity was no longer independently associated with dementia incidence. Compared with Israeli-born Jewish patients, the adjusted subdistribution hazard ratios were 1.03 (95% CI: 0.91–1.17) for Arab patients, 1.05 (95% CI: 0.76–1.45) for Ethiopian patients, and 0.99 (95% CI: 0.91–1.07) for former Soviet Union-born patients. Older age, male sex, cardiovascular disease, and lower socioeconomic status remained independently associated with a higher subdistribution hazard of dementia.

### Age at dementia diagnosis and clinical differences

The mean age at dementia diagnosis was highest among the Ethiopian group and lowest among the Arab group (75.8 ± 9.5 vs 70.3 ± 7.4 years; *P* < 0.001).

In R and H, women develop dementia at a slightly older age than men (73.1 vs 72.1 in the H group, 75.1 vs 76.2 in the E group). In the R and A groups, the opposite is true (70.1 vs 70.4 in the A group and 74.5 vs 74.1 in the R group). The difference is small (0.5–1.5 years) but statistically significant.

Among all groups, E patients demonstrated the best kidney function, reflected by the highest mean GFR levels, and had a lower prevalence of comorbidities. In contrast, Russian-origin patients had the highest burden of comorbidities, including cardiovascular disease, CKD, and heart failure. Female sex predominated in both the R and A groups (Table 1).

**Table 1:** Demographic and anthropomorphic characteristics of patients.

	Total	A	E	H	R	*P*-value
Number of patients	*n* = 150,237	*n* = 18,137	*n* = 2,208	*n* = 99,998	*n* = 29,894	
Dementia *n* (%)	*n* = 58,728 (39%)	*n* = 5,854 (32%)	*n* = 693 (31%)	*n* = 39,559 (40%)	*n* = 12,590 (42%)	0.001
Age at event (M)	73 ± 8.3	70.4 ± 7.5	76 ± 9.7	72.2 ± 8	74.3 ± 8.3	0.001
Age et event (F)	73 ± 8.1	70.2 ± 7.4	75 ± 9	73 ± 8	75 ± 8	0.001
Gender: male *n* (%)	70,287 (47%)	7,530 (41%)	1,080 (49%)	49,529 (49%)	12,148 (40%)	0.001
Gender: female *n* (%)	79,950 (53%)	10,607 (59%)	1,128 (51%)	50,469 (51%)	17,746 (60%)	0.001
Socioeconomic (high)	46,352 (31%)	366 (1.6%)	144 (6%)	44,037 (37%)	7,473 (25%)	0.001
Female	25,617 (28%)	196 (1.6%)	74 (6%)	20,703 (36%)	4,644 (23%)	0.001
Male	27,481 (33%)	170 (1.9%)	70 (6%)	23,334 (40%)	6,165 (43%)	
Medium	56,740 (38%)	2,405 (13%)	754 (34%)	40,707 (41%)	12,874 (43%)	0.001
Medium	34,471 (38%)	1,624 (13.3%)	440 (36%)	23,678 (40.8%)	8,729 (43%)	0.001
Female/male	31,195 (38%)	1,133 (12.7%)	390 (32.9%)	23,507 (40.1%)	6,165 (43%)	
Low	47,145 (31%)	15,432 (85%)	1,318 (60%)	20,848 (21%)	9,547 (32%)	0.001
Low	31,507 (34%)	10,271 (85%)	711 (58%)	13,642 (23.5%)	6,883 (34%)	0.001
Female/male	24,484 (29%)	7,619 (85%)	727 (62%)	11,842 (20.2%)	4,296 (29.9%)	
Smoking (current)	13,853 (11%)	2,546 (16%)	40 (2%)	9,370 (12%)	1,897 (8%)	0.001
Former	27,978 (23%)	2,749 (18%)	50 (3%)	20,574 (25%)	4,605 (19%)	0.001
Never	81,264 (66%)	10,418 (66%)	1,708 (95%)	51,612 (63%)	17,526 (73%)	0.001
BMI (kg/m^2^)	29 ± 5.3	30.5 ± 5.9	25.7 ± 4.3	28.5 ± 5.3	29.5 ± 5.6	0.001
HbA1c (%)	7.1 ± 1.4	7.4 ± 1.6	7.5 ± 1.8	7.1 ± 1.4	7.0 ± 1.3	0.001
GFR mL/min	66 ± 23	68 ± 23	81 ± 20	66 ± 22	62 ± 22	0.001
Cardiovascular *n* (%)	108,669 (72.3%)	12,321 (67%)	1,116 (51%)	73,018 (73%)	22,214 (74%)	0.001
Congestive heart *n* (%)	57,477 (38%)	6,850 (38%)	512 (23%)	38,118 (38%)	11,997 (40%)	0.001
Kidney disease	74,959 (50%)	8,436 (47%)	552 (25%)	50,012 (50%)	15,959 (53%)	0.001
Hypertension	140,631 (93.6%)	16,934 (93.4%)	1,905 (86.3%)	92,896 (92.9%)	28,896 (96.7%)	0.001

Prescription patterns for six major classes of antidiabetic medications, including metformin, short-acting and long-acting insulin, DPP-4 inhibitors, SGLT-2 inhibitors, GLP-1 receptor agonists, and sulfonylureas (SFUs), were analyzed. Overall, metformin was prescribed to 65% of patients, SFUs to 28%, GLP-1 receptor agonists to 23%, long-acting insulin to 22%, DPP-4 inhibitors to 19%, and SGLT-2 inhibitors to 13% (Table 2).

**Table 2:** Antidiabetic and comorbidity-related medications.

	Total	A	E	H	R	*P*-value
Medication (*n*, %)	*n* = 150,237	*n* = 18,137	*n* = 2,208	*n* = 99,988	*n* = 29,894	*
Metformin	97,698 (65%)	12,780 (71%)	1,459 (66%)	64,906 (65%)	18,553 (62%)	*
GLP-1	33,909 (23%)	6,003 (33%)	480 (22) %	21,611 (22%)	5,815 (19.5%)	*
SGLT-2	19,757 (13%)	2,688 (15%)	186 (8.4%)	13,609 (14%)	3,274 (11.0%)	*
DPP-4	28,828 (19%)	2,199 (12%)	153 (7%)	21,613 (22%)	4,863 (16%)	*
Insulin – long	32,708 (22%)	6,062 (33%)	484 (22%)	20,507 (20.5%)	5,655 (19%)	*
Insulin – short	14,522 (10%)	3,020 (17%)	145 (6.6%)	8,862 (9%)	2,495 (8.3%)	*
SFUs	41,593 (28%)	4,521 (25%)	643 (29%)	27,459 (28%)	8,970 (30%)	*
Statins	121,287 (81%)	15,183 (84%)	1,429 (65%)	80,670 (81%)	24,005 (80.3%)	*
ACE/ARB	107,171 (71%)	12,994 (72%)	1,345 (61%)	70,262 (70.3%)	22,570 (75.5%)	*

* PV < 0.001 for all.

**Figure 2:**
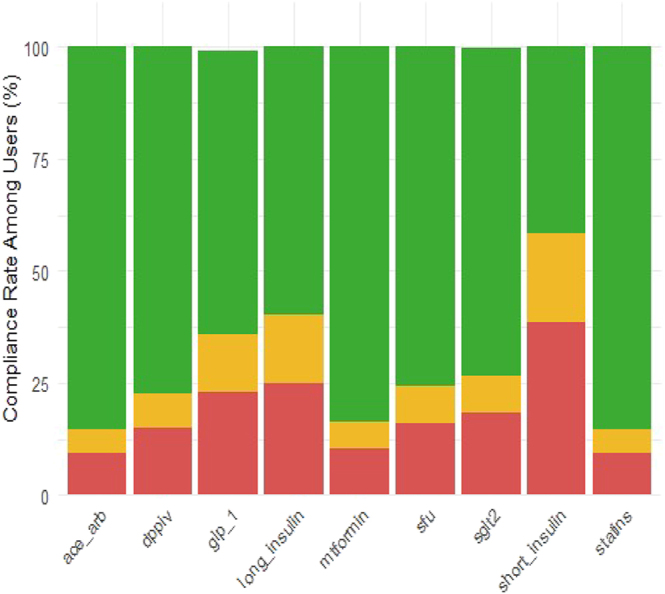
Medication compliance among patients.

Compliance with oral antidiabetic medications was higher than with injectables. It was highest with metformin among all groups (84% of patients across all groups; 78% in group E) and DPP-4 inhibitors (77% in groups R and H, 75% in group A, and 66% in group E). Compliance with long-acting insulin was observed in 60% of patients, with the highest rate in group R and the lowest in group E (64 vs 50%). Compliance with GLP-1 was highest in group R and lowest in group E (67.2 vs 48%); compliance with SGLT-2 inhibitors was highest in group H (74%) and group R (72%) and lowest in group E (62%) (Fig. 2).

**Figure 3:**
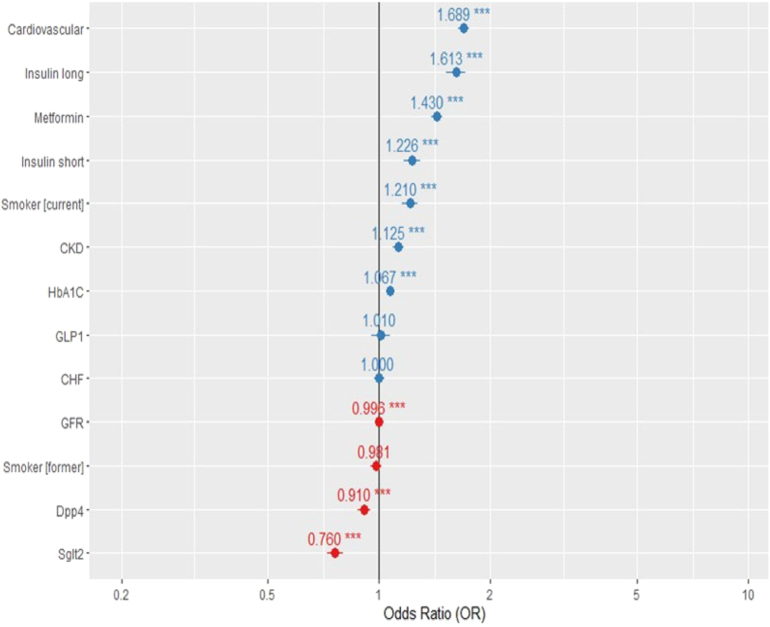
Conditional logistic regression showing odds ratios (ORs) for dementia probability in the matched cohort.

Conditional logistic regression revealed that SGLT-2 and DPP-4 inhibitors were associated with a lower probability of dementia (OR 0.76; 95% CI: 0.72–0.79; OR 0.80, 95% CI: 0.79–0.85, respectively), while cardiovascular disease (OR 1.69) treatment with insulin and metformin (OR 1.5; 95% CI: 1.52–1.72) was associated with a higher probability (OR 1.25, 95% CI: 1.13–1.37; OR 1.62, 95% CI: 1.57–1.69) (Fig. 3).

**Figure 4:**
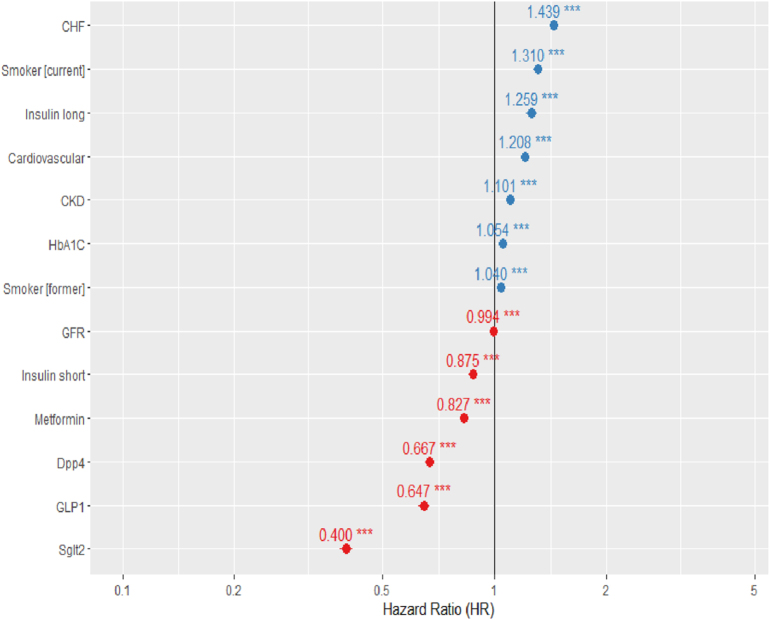
Cox proportional hazards regression of predictors of dementia-related mortality.

In Cox proportional hazards models, treatment with SGLT-2 inhibitors (HR 0.38, 95% CI: 0.36–0.40), GLP-1 receptor agonists (HR 0.64, 95% CI: 0.61–0.66), and DPP-4 inhibitors (HR 0.67, 95% CI: 0.65–0.68) was associated with a significantly reduced risk of dementia-related mortality. Cardiovascular disease, kidney disease, and CHF were associated with an increased probability of dementia-related death (HR 2.1, 95% CI: 1.34–3.39; HR 1.74, 95% CI: 1.27–2.3; HR 1.48, 95% CI: 1.14–1.92) (Fig. 4).

## Discussion

The study examined the relationship between ethnicity, type 2 diabetes, and the risk of developing dementia, with a particular focus on patterns of antihyperglycemic medication use. Although 39% of participants were diagnosed with dementia over the 20-year study period, this crude proportion primarily reflects the long duration of follow-up and the advanced age of the cohort. When dementia incidence was estimated using a Fine–Gray competing-risk model, the 10-year cumulative incidence was approximately 9–10%, increasing to 36–39% over 20 years. Notably, the highest 10-year cumulative incidence was observed among Ethiopian-born patients (13.8%), followed by Arab patients (10.5%). In contrast, Israeli-born Jewish and former Soviet Union-born individuals demonstrated lower cumulative incidence (8.8 and 8.6%, respectively), with Russian-origin patients exhibiting the lowest cumulative incidence function (CIF).

Our findings are consistent with previous population-based studies. Moran *et al.* reported dementia incidence rates of 10.2 per 1,000 person-years among individuals with well-controlled HbA1c (6–7%) and 19.3 per 1,000 person-years among those with poorly controlled HbA1c (≥10%) in the Northern California Kaiser Permanente cohort (17). Similarly, a marked increase in dementia risk was demonstrated with advancing age and diabetes duration, reaching approximately 23 cases per 1,000 person-years among individuals aged over 80 years (18). Other large cohorts from Denmark, Australia, and the United States have reported cumulative dementia proportions of 15–25% after 10–15 years of follow-up (19, 20). Previous studies have estimated dementia prevalence in the Arab population in Israel at 10–20%, potentially higher than that observed in the Jewish population (21). While unadjusted analyses in our study also demonstrated ethnic differences in cumulative dementia incidence, these differences were substantially attenuated after multivariable adjustment. In Fine–Gray competing-risk regression models accounting for age, sex, socioeconomic status, cardiovascular disease, CKD, and CHF, ethnicity was no longer independently associated with dementia incidence, and sex did not emerge as a significant predictor. Together, these findings suggest that the observed ethnic- and sex-related differences in unadjusted cumulative incidence are largely attributed to variations in comorbidity burden, survival patterns, and competing mortality, rather than to intrinsic ethnic or biological susceptibility to dementia.

Compliance with prescribed medications in our study ranged from 60 to 80% for most antidiabetic drugs, except for short-acting insulin, where it dropped below 50%. Among all ethnic groups, Ethiopian-origin patients showed the lowest compliance. A large meta-analysis of 156 studies (22) indicated that the pooled proportion of adherent patients was 54% (95% CI: 51–58%) and was unaffected by the length of the observation period (<12 months or >12 months). However, adherence was highest among oral antihyperglycemics for SGLT-2, TZD, and DPP-4 medications. In our study, treatment with metformin and DPP-4 inhibitors demonstrated the highest adherence, likely because of the lower cost of the former and the lack of adverse reactions associated with the latter. A relatively small proportion of our patients received new classes of antidiabetic medications, with 23% prescribed GLP-1 agonists and 13% SGLT-2 inhibitors. According to other real-world data (23, 24), less than 15% of patients with cardiovascular risk factors and CKD have prescriptions for GLP-1 and SGLT-2 inhibitors. In a recent study (25), a multidisciplinary quality improvement intervention significantly increased prescribing of SGLT-2 inhibitors and GLP-1 receptor agonists.

Aside from solid evidence of renal outcomes and cardiovascular protection by GLP-1 and SGLT-2 treatments (26, 27), and improvements in blood glucose control, their potential neuroprotective effect has been reported in several studies. In our study, we found a significant effect of SGLT-2 inhibitors on reducing the probability of dementia and dementia-related death even after adjustment for potential confounders, including age, HbA1c, BMI, comorbidities, and other relevant factors.

Interestingly, DPP-4 inhibitors, which were not found to increase or decrease cardiovascular outcomes (28), were shown in animal (29) and some small human studies to be neuroprotective and to improve insulin resistance (30). Consistent with these findings, we observed that DPP-4 inhibitors were associated with a reduced risk of developing dementia, whereas GLP-1 was linked to dementia-related death but not to the likelihood of developing dementia.

Several studies have shown that metformin is related to a small but significant risk reduction in dementia development (31). In contrast to these findings, in our study, metformin treatment was not associated with the same findings.

Treatment with SGLT-2 inhibitors, DPP-4, and GLP-1 receptor agonists was significantly associated with a reduced risk of dementia-related mortality. In both the overall and adherence-restricted analyses, SGLT-2 inhibitors demonstrated a robust protective effect. These findings support the potential neuroprotective role of newer antidiabetic therapies.

## Conclusion

In this large, population-based cohort of older adults with type 2 diabetes, the use of newer antihyperglycemic therapies was differentially associated with dementia outcomes. Treatment with SGLT-2 inhibitors and DPP-4 inhibitors was associated with a lower probability of developing dementia in matched analyses. SGLT-2 inhibitors, GLP-1 receptor agonists, and DPP-4 inhibitors were associated with a significantly reduced risk of dementia-related mortality in time-to-event analyses. Although ethnic differences in dementia incidence were observed in unadjusted analyses, these associations were largely attenuated after multivariable competing-risk adjustment, suggesting that comorbidity burden and competing mortality, rather than intrinsic ethnic susceptibility, primarily explain the observed differences. Collectively, these findings support a potential role for selected newer antihyperglycemic agents – particularly SGLT-2 inhibitors – in modifying long-term neurodegenerative outcomes among patients with type 2 diabetes.

### Study limitations

Prescription refill data may not accurately represent actual medication use. While the use of pharmacy dispensation records minimizes misclassification from unfilled prescriptions, adherence beyond pharmacy refills cannot be fully verified.

Confounding by indication and unequal access to new antidiabetic therapies among different ethnic groups could affect the observed relationships. Since exposure was defined within a two-year window before the index date, and given that dementia symptoms may precede clinical diagnosis by up to a decade, our findings mainly reflect recent medication use, while longer cumulative exposure may not have been fully captured, potentially leading to underestimation of long-term protective or harmful effects. We did not perform a dedicated analysis of complex combination therapy patterns (e.g., ≥2 concurrent drug classes), which may have different clinical effects. More detailed future analyses are warranted.

In addition, unmeasured socioeconomic or cultural factors may influence dementia diagnosis rates or healthcare-seeking behaviors, especially in minority populations. We did not perform a dedicated analysis of complex combination therapy patterns (e.g., ≥2 concurrent drug classes), which may have different clinical effects. More detailed future analyses are warranted.

Despite these challenges, the consistent findings across sensitivity analyses suggest that the protective association with SGLT-2 inhibitors and GLP-1 receptor agonists on dementia-related mortality remains robust.

## Declaration of interest

The authors declare that there is no conflict of interest that could be perceived as prejudicing the impartiality of the work reported.

## Funding

This work did not receive any specific grant from any funding agency in the public, commercial, or not-for-profit sector.

## Ethical considerations

This study was conducted in accordance with the principles of the Declaration of Helsinki and was approved by the Clalit Health Services Institutional Review Board (IRB approval number: EMC-0117-24). The study used fully de-identified data extracted from the Clalit electronic health records, and no direct patient contact occurred. Therefore, the ethics committee waived the requirement for informed consent. Data confidentiality and patient privacy were strictly maintained throughout all stages of data handling, storage, and analysis.
